# Residential Segregation and Lung Cancer Risk in African American Adults

**DOI:** 10.1001/jamanetworkopen.2025.18481

**Published:** 2025-07-01

**Authors:** Yi Xiao, Xiaoke Zou, Calvin P. Tribby, Peter Vien, Christina Chang, Richard J. Curley, Olutola Akande, Melinda C. Aldrich, Sophia Wang, Rick A. Kittles, Kimlin Ashing, F. Lennie Wong, Marta M. Jankowska, Tarik Benmarhnia, Loretta Erhunmwunsee

**Affiliations:** 1 Department of Population Sciences, City of Hope Comprehensive Cancer Center, Duarte, California; 2 Department of Surgery, City of Hope Comprehensive Cancer Center, Duarte, California; 3 Department of Thoracic Surgery, Vanderbilt University Medical Center, Nashville, Tennessee; 4 Computational and Quantitative Medicine, City of Hope Comprehensive Cancer Center, Duarte, California; 5 Department of Community Health and Preventive Medicine, Morehouse School of Medicine, Atlanta, Georgia; 6 Scripps Institution of Oceanography–Climate, Atmospheric Sciences, Physical Oceanography, San Diego, California; 7 Irset Institut de Recherche en Santé, Environnement et Travail, UMR-S 1085, Inserm, University of Rennes, EHESP, Rennes, France; 8 Division of Epidemiology, Vanderbilt University Medical Center, Nashville, Tennessee

## Abstract

**Importance:**

Although structural racism as manifested by residential segregation is a documented root cause of race-based disparities in lung cancer survival and care, its impact on lung cancer development remains underexplored.

**Objective:**

To examine the association between residential segregation and lung cancer incidence and to identify modifiable factors mediating this association.

**Design, Setting, and Participants:**

This cohort study used data from the Southern Community Cohort Study (SCCS), which enrolled African American and non-Hispanic White participants who had no cancer at enrollment. The SCCS recruited participants from community health centers and random sampling from 12 southeastern states. Follow-up occurred between March 2002 to December 2019. Analysis was performed from April 2022 to March 2025.

**Exposures:**

Residential segregation, measured by the isolation index using 2010 census block group data, was linked to participants’ baseline address.

**Main Outcomes and Measures:**

Incident lung cancer cases were identified via state cancer registries and the National Death Index (December 31, 2016, to December 31, 2019, depending on the state). Parametric g-computation estimated cumulative lung cancer risk under hypothetical interventions reducing residential segregation. Mediation analyses utilized inverse propensity weighting and marginal structural models to assess potential mediating pathways.

**Results:**

The cohort comprised 71 634 participants (median [IQR] age, 50 [45-57] years; 42 032 [58.7%] female; 50 898 [71.1%] African American and 20 736 [28.9%] non-Hispanic White) enrolled between 2002 and 2009. All hypothetical scenarios of lowering the isolation index were associated with lower 17-year culminative incidence of lung cancer among African American individuals but not among non-Hispanic White individuals. Approximately 24.7% (95% CI, 17.1%-36.6%) of the association between residential segregation and lung cancer risk in African American participants was mediated by menthol smoking, 13.1% (95% CI, 3.2%-25.4%) by exposure to particulate matter with a diameter of 2.5 μm or less, 4.7% (95% CI, 1.3%-9.6%) by secondhand smoke exposure at home, and 4.6% (95% CI, 2.1%-7.7%) by education.

**Conclusions and Relevance:**

In this cohort study of non-Hispanic White and African American individuals, lower residential segregation was significantly associated with decreased lung cancer risk for African American individuals but not their non-Hispanic White counterparts. Mediation analysis identified mentholated smoking use and air pollution exposure as 2 major pathways between residential segregation and lung cancer incidence among African American adults. These findings suggest that lung cancer development reduction efforts should include policies with targetable segregation metrics and initiatives that lower exposure to documented mediators.

## Key Points

**Question:**

To what extent is lung cancer risk among African American adults associated with residential segregation and its mediating factors?

**Findings:**

In this cohort study with 71 634 participants, reduced residential segregation was significantly associated with fewer lung cancer cases among African American but not non-Hispanic White adults. Mediation analysis identified several factors mediating the association, including mentholated smoking, air pollution, education, and secondhand smoke exposure.

**Meaning:**

This study suggests that structural racism embedded in neighborhood conditions contributes to lung cancer development and provides evidence for policymakers and public health leaders working to reduce disparities.

## Teaser

This cohort study examines whether residential segregation is associated with lung cancer incidence between African American and non-Hispanic White adults in the US South and whether any modifiable factors mediate this association.

## Introduction

Lung cancer is the number one cancer killer in the United States. Non-Hispanic Black and African American individuals in the United States develop lung cancer 5 years earlier^1^ and have higher lung cancer mortality rates^2^ than their non-Hispanic White counterparts. African American individuals have the highest lung cancer incidence among all populations residing in the southern US states.^3^ Factors beyond tobacco, including socioeconomic conditions, significantly contribute to these disparities; however, the impact of racial residential segregation—a critical structural determinant of health—on African American lung cancer risk is underexplored.^4,5,6^ The mechanisms linking residential segregation to African American lung cancer risk also remain poorly understood.^5,7,8^

Residential segregation is an expression of contemporary structural racism that continues to negatively impact the health of African American individuals today.^9^ African American people are often excluded from prosperous communities, resulting in neighborhoods that are frequently deprived and exposed to traffic and toxic waste.^10,11,12^ Along with historical redlining, processes like White flight, urban renewal, disinvestment, subprime lending, the foreclosure crisis, gentrification, and displacement have all added to the modern marginalization and isolation of African American neighborhoods.^13^ Particularly in the US South, during the Great Migration, residential segregation intensified as African American people moved from rural areas into southern cities and White residents relocated to suburbs, resulting in segregated urban communities.^14^ Numerous studies link segregation to higher African American lung cancer mortality,^15,16,17^ but the specific impact of segregation and its mediating factors on lung cancer risk remain insufficiently understood, limiting our understanding of segregation’s role in the etiology of lung cancer in African American individuals.

Residential segregation encompasses multiple dimensions that capture the interaction of subgroups within a geographical area, including unevenness, isolation, centralization, concentration, and clustering.^18^ Various indices measure segregation, including the dissimilarity index, which is frequently used but has limitations, including that it ignores the relative size of the groups being compared.^17,19^ In contrast, the isolation index, which measures the degree to which members from a group interact exclusively with others from the same group, explicitly accounts for relative group sizes, a property that has been previously leveraged in lung cancer outcomes work^17^ and was used in the present study.

Causal mediation analysis helps identify the pathways through which residential segregation affects lung cancer incidence, which is pertinent given that racial segregation increases the exposure to many carcinogens, including air pollutants, like particulate matter with a diameter of 2.5 µm or less (PM_2.5_) and nitrogen dioxide.^20,21^ Additionally, racial segregation is linked to higher tobacco retailers and marketing to African American communities,^22,23^ and there is clear evidence of the systemic exploitation of racial markets in the sale and distribution of menthol cigarettes, specifically, which African American individuals who smoke overwhelmingly consume even now.^24^ The tobacco industry’s targeted racial marketing is one of many ways that structural racism influences lung cancer risk, but the interplay between smoking, air pollution exposure, segregation, and lung cancer risk has been understudied. This study will investigate, for the first time that we are aware of, the association of residential segregation with lung cancer incidence among African American and non-Hispanic White individuals in southern US states and assess the role of prespecified mediators in this association.

Our theoretical approach and proposed causal pathway between residential segregation and lung cancer risk builds on the multilevel conceptual framework described by Alcaraz et al, who state that “racial residential segregation, a type of structural discrimination in housing that engenders inequitable access to social and economic resources, is a root cause of black-white racial disparities in health.”^25^ Based on this model and the literature, we propose that residential segregation influences laws, housing regulations, and policies that create economic (household income, education, and employment) and physical (PM_2.5_ exposure, primary and secondhand smoke exposure) living environments that increase risk of lung cancer.

## Methods

### Study Participants

The Southern Community Cohort Study (SCCS) enrolled nearly 86 000 English-speaking individuals aged 40 to 79 years between March 2002 and December 2009 from 12 southern states (eMethods in Supplement 1).^26,27^ Approximately 86% of participants were recruited from community health centers serving medically underserved populations, with the remainder selected via random sampling from the general population. At baseline, participants provided sociodemographic, lifestyle, medical background, and food dietary information. Three follow-ups occurred at 5-year intervals to update health and lifestyle data. Follow-up mortality data were determined through annual linkage to the Social Security Administration and the National Death Index. Written informed consent was obtained, and the SCCS was approved by the institutional review boards of Vanderbilt University Medical Center and Meharry Medical College. The study follows the Strengthening the Reporting of Observational Studies in Epidemiology (STROBE) reporting guidelines.

Eligible participants were self-identified as non-Hispanic African American (African American hereafter) or non-Hispanic White based on options provided by investigators, had no cancer diagnosis at enrollment, and resided in areas with a valid isolation index. Individuals from other racial and ethnic groups, including American Indian or Alaska Native, Asian or Pacific Islander, Hispanic or Latino, multiracial, and other, were excluded due to small sample size. We included race and ethnicity in the analysis due to their reported association with lung cancer incidence. Overall, 71 634 SCCS participants meeting the inclusion criteria were included in the current study.

### Outcomes

Incident lung cancer cases (*International Classification of Disease for Oncology, Third Edition* diagnosis codes C340-C349) were determined through state cancer registries and/or National Death Index mortality records.^27^ Time to lung cancer was calculated from enrollment to lung cancer diagnosis date, with censoring at death, loss to follow-up, or the end of the follow-up period (ranging from December 31, 2016, to December 31, 2019, depending on the state).

### Exposure

The Isolation index measures the extent to which a group is isolated from other groups within a geographical area (see formula in eMethods in Supplement 1).^28^ This index ranges from 0 to 1, with higher scores indicating greater isolation. In this study, we measured isolation index for African American individuals, and an index of 1 suggests complete isolation, where all African American individuals reside in a single block, and lower values indicate more integration. An isolation index of 0.7 or greater is regarded as highly segregated.^5^ Census block calculations were based on Census 2010 SF1 P3 data linked to participants’ baseline addresses.

### Individual-Level Covariates

Covariates included self-reported employment, race, ethnicity, education, income, tobacco use, age, sex, and secondhand smoke exposure and were collected at baseline.^26,27,29^ Categories used in Table 1 were applied, with dichotomization of education (ie, less than college vs some college or more) and household income (less than $25 000 vs $25 000 and greater) in mediation analysis. Missing covariate data (<4%) were imputed using medians or modes.

**Table 1.** Demographic Characteristics of the Southern Community Cohort Study

Characteristic	Participants, No. (%)			*P* value^a^
	Overall (N = 71 634)	Non-Hispanic White (n = 20 736)	African American (n = 50 898)	
Lung cancer cases	1727 (2.4)	566 (2.7)	1161 (2.3)	<.001
Isolation Index, median (IQR)	0.7 (0.3-0.9)	0.2 (0.04-0.4)	0.8 (0.6-0.9)	<.001
PM_2.5_, mean (SD), μg/m^3^	11.2 (1.8)	10.8 (2.0)	11.2 (1.8)	<.001
NA	325	195	130	NA
Sex				
Female	42 032 (58.7)	12 561 (60.6)	29 471 (57.9)	<.001
Male	29 602 (41.3)	8175 (39.4)	21 427 (42.1)	
Smoking status				
Never smoking	25 817 (36.0)	6943 (33.5)	18 874 (37.1)	<.001
Primarily menthol smoking	30 319 (42.3)	3613 (17.4)	26 706 (52.5)	
Primarily nonmenthol smoking	15 266 (21.3)	10 102 (48.7)	5164 (10.1)	
Not reported	232 (0.3)	78 (0.4)	154 (0.3)	
Age, median (IQR), y	50 (45-57)	52 (46-59)	50 (45-56)	<.001
Educational attainment				
<9 y	5687 (7.9)	1674 (8.1)	4013 (7.9)	<.001
High school or less than college	43 534 (60.8)	11 191 (54.0)	32 343 (63.5)	
College and more	22 372 (31.2)	7861 (37.9)	14 511 (28.5)	
Not reported	41 (0.1)	10 (<0.1)	31 (0.1)	
Household income attainment				
<$15 000	40 004 (55.8)	9831 (47.4)	30 173 (59.3)	<.001
≥$15 000 to <$25 000	15 147 (21.1)	3862 (18.6)	11 285 (22.2)	
≥$25 000 to <$50 000	9578 (13.4)	3337 (16.1)	6241 (12.3)	
≥$50 000	6007 (8.4)	3418 (16.5)	2589 (5.1)	
Not reported	898 (1.3)	288 (1.4)	610 (1.2)	
Current employment status				
Unemployed	42 443 (59.2)	12 304 (59.3)	30 139 (59.2)	.004
Employed	28 142 (39.3)	8177 (39.4)	19 965 (39.2)	
Not reported	1049 (1.5)	255 (1.2)	794 (1.6)	
Secondhand smoke exposure at home				
No	45 145 (63.0)	13 005 (62.7)	32 140 (63.1)	<.001
Yes	24 026 (33.5)	6843 (33.0)	17 183 (33.8)	
Not reported	2463 (3.4)	888 (4.3)	1575 (3.1)	
Secondhand smoke exposure at other places				
No	42 411 (59.2)	12 729 (61.4)	29 682 (58.3)	<.001
Yes	26 453 (36.9)	7002 (33.8)	19 451 (38.2)	
Not reported	2770 (3.9)	1005 (4.8)	1765 (3.5)	

Abbreviations: NA, not applicable; PM_2.5_, particulate matter with a diameter of 2.5 μm or less.

^a^ Pearson χ^2^ test for categorical variables; Wilcoxon rank sum test for continuous variables.

### Neighborhood-Level Covariates

Annual exposure to PM_2.5_ was estimated using the historical PM_2.5_ across North America dataset (1981 to 2016).^30^ Yearly mean PM_2.5_ per census tract was computed using raster points, with missing values imputed from the nearest census tract. The participant demographic characteristics reflected the 10-year average PM_2.5_ exposure until lung cancer diagnosis or censoring. In parametric g-computation and mediation analysis, a 5-year moving average of PM_2.5_ was used.

### Statistical Analysis

Baseline characteristics were described with median (IQR) or mean (SD) for continuous variables and frequency (percentage) for categorical variables. A χ^2^ test or Wilcoxon rank sum test was conducted to examine the differences in baseline variables. Two-sided *P* < .05 indicated statistical significance. Geographical clustering was not considered due to low heterogeneity across the states.

#### Parametric G-Computation

Using the potential outcomes framework, we assumed exchangeability, positivity, and consistency. We included predefined confounders and managed positivity using quartiles of the segregation index. Regarding consistency, we considered modifiable mediators proposed to quantify the drivers of health inequities.^31^ Parametric g-computation, a flexible causal inference method used in epidemiology, was employed for its ability to simulate real-world exposure scenarios, handle time-varying exposures, and provide intuitive effect estimates.^31,32,33,34^

We estimated the cumulative lung cancer risk associated with segregation by comparing hypothetical isolation index values with observed values. We adjusted for household income, education, smoking, employment status, age, sex, secondhand smoking exposure, and a time-varying covariate (5-year moving average of PM_2.5_). Two hypothetical interventions were tested: reducing the isolation by a fixed percentage annually or to specific thresholds (first quartile, median, third quartile). An isolation index below the threshold remained unchanged. The natural course (most segregated) served as the reference. Details of parametric g-computation were described elsewhere.^35^ Bootstrapping (100 samples) estimated 95% CIs. Cochran *Q* tests assessed heterogeneity between African American and non-Hispanic White participants.

#### Mediation Analysis

**Figure 1.**
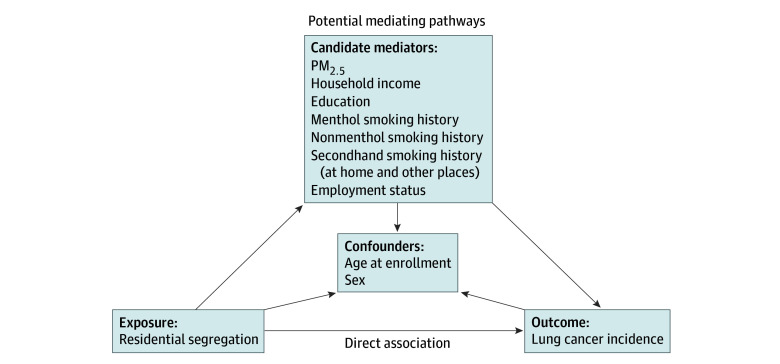
A Simplified Diagram Depicting the Associations Between Residential Segregation and Lung Cancer Incidence PM_2.5_ indicates particulate matter with a diameter of 2.5 μm or less.

We conducted a mediation analysis to estimate the proportion of the association between residential segregation and lung cancer incidence that may be explained by potential mediators (indirect pathway), with the remaining reflecting the direct association (Figure 1). Variables with reported association with lung cancer risk and with association to residential segregation were assessed as potential mediators, including PM_2.5_ exposure, household income, education, smoking status, unemployment, and secondhand smoke exposure.^1,5,36^ Smoking status was categorized into menthol and nonmenthol use. The role of mediators was assessed individually using marginal structural models (MSMs) with inverse propensity weighting (IPW).^37^ Additionally, inverse odds ratio weighting (IORW) analyzed the joint effect of all mediators.^38^ Both models included sex and age as confounders. Total, direct, and indirect effects were quantified, with confidence intervals obtained through bootstrapping. Further details are described in eMethods in Supplement 1.

All statistical analyses were performed using the R version 4.0.3 (R Project for Statistical Computing). We considered 95% CIs excluding 0 statistically significant.

## Results

Among 71 634 participants, 1727 (2.4%) were diagnosed with lung cancer since enrollment, and 25 817 (36.0%) never smoked. Overall, 42 032 (58.7%) were female, and 29 602 (41.3%) were male. The median (IQR) age was 50 (45-57) years, with 20 736 (28.9%) non-Hispanic White and 50 898 (71.1%) African American. Median (IQR) follow-up was 12.5 (10.0-14.4) years, with a maximum follow-up of 17 years, for which the 17-year cumulative risk was assessed. Mean (SD) 10-year average PM_2.5_ exposure before lung cancer diagnosis or censoring was 11.2 (1.8) μg/m^3^ among African American participants and 10.8 (2.0) μg/m^3^ among non-Hispanic White participants. The median (IQR) isolation index was 0.8 (0.6-0.9) for African American participants and 0.2 (0.04-0.4) for non-Hispanic White participants. For participants of both races, higher PM_2.5_ and menthol and nonmenthol smoking were associated with increased lung cancer risk, while higher income and education were associated with lower risk (eTable 1 in Supplement 1). For both racial groups, the proportion of mentholated usage increased in the more segregated group. However, among African American participants, nonmentholated smoking decreased with increasing segregation (eTables 2 and 3 in Supplement 1).

**Figure 2.**
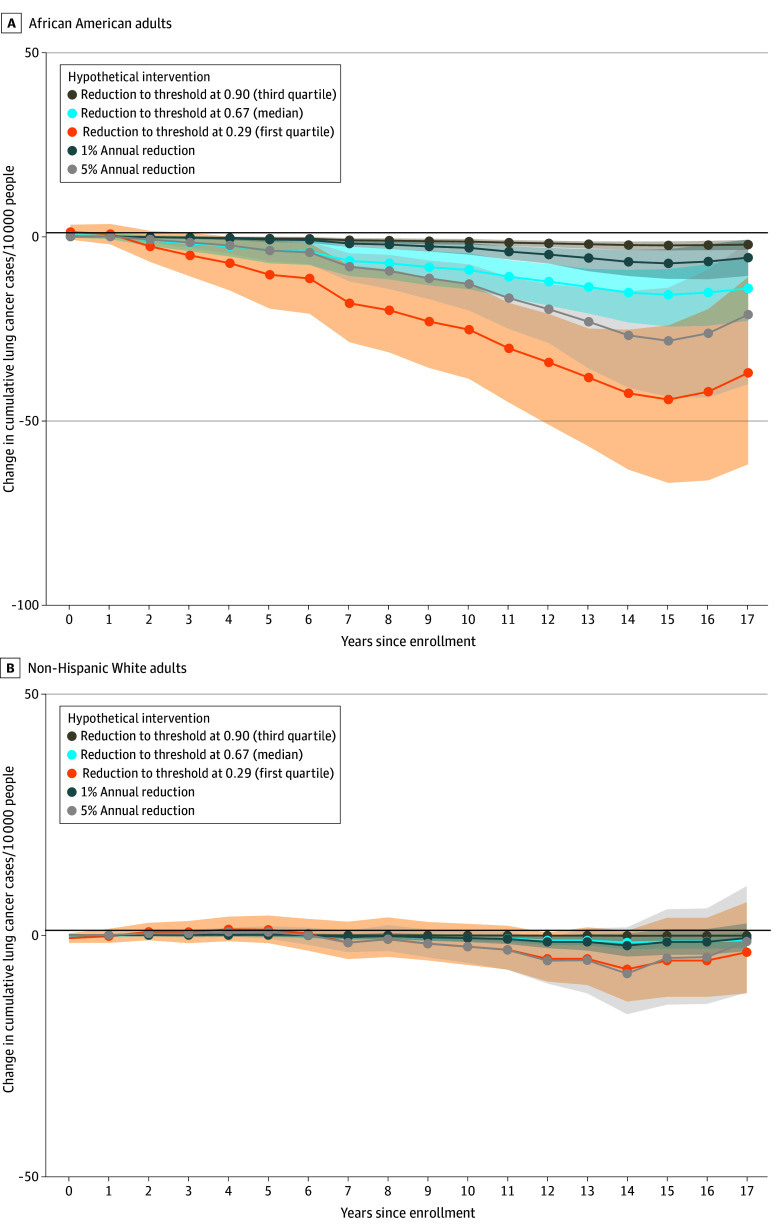
Change in Cumulative Lung Cancer Cases Associated With Strategies Reducing Isolation Index

Among African American participants, all hypothetical strategies lowering the isolation index were associated with a lower number of lung cancer cases compared with the natural course by the 17th year of follow-up (Figure 2A and Table 2). Among African American individuals, reducing the isolation index to the first quartile (least segregation, isolation index: 0.29) was associated with a reduction of 36.99 (95% CI, 11.03–61.81) lung cancer cases per 10 000 individuals. A reduction to the median isolation index (0.65) was associated with a decrease of 14.08 (95% CI, 5.53–22.87) cases per 10 000 individuals, while a reduction to the third quartile (0.90) was associated with a decrease of 2.21 (95% CI, 0.97–3.63) cases per 10 000 individuals. Similarly, a 1% yearly reduction in isolation was associated with a decrease of 5.75 (95% CI, 0.81-10.70) lung cancer cases per 10 000 individuals in African Americans, while a 5% yearly reduction was associated with a decrease of 21.20 (95% CI, 1.65-40.08) cases per 10 000 individuals. Among non-Hispanic White participants, incidence ratios associated with isolation index decrease were not significant, as indicated by 95% CIs including 1 (Figure 2B). The Cochran *Q* test suggested heterogeneity between races (*P* < .001).

**Table 2.** Comparing Estimated 17-Year Cumulative Risk of Lung Cancer Under Different Thresholds Compared With Natural Course^a^

Intervention strategy	Estimate (95% CI)			
	Among African American individuals		Among non-Hispanic White individuals	
	Reduced lung cancer cases, No. per 10 000 people	Reduction in lung cancer risk, %	Reduced lung cancer cases, No. per 10 000 people	Reduction in lung cancer risk, %
Natural course	0 [Reference]	0 [Reference]	0 [Reference]	0 [Reference]
Threshold value				
0.90 (More segregated)	2.21 (0.97 to 3.63)	0.67 (0.29 to 1.11)	0.09 (−0.02 to 0.22)	0.02 (0 to 0.05)
0.65	14.08 (5.53 to 22.87)	4.26 (1.64 to 6.96)	1.07 (−0.54 to 2.67)	0.26 (−0.13 to 0.63)
0.29 (Less segregated)	36.99 (11.03 to 61.81)	11.19 (3.33 to 19.05)	3.51 (−6.81 to 12.01)	0.85 (−1.59 to 2.9)
Annual reduction, %				
1	5.75 (0.81 to 10.7)	1.74 (0.24 to 3.25)	0.58 (−2.43 to 3.27)	0.14 (−0.57 to 0.8)
5	21.2 (1.65 to 40.08)	6.42 (0.5 to 12.19)	1.3 (−10.14 to 11.85)	0.33 (−2.38 to 2.83)

^a^ All models were adjusted by time-varying variable, exposure to particulate matter with a diameter of 2.5 μm or less, and time-fixed variables, sex, enrollment age, educational attainment, household income, menthol and nonmenthol smoking history, unemployment, and secondhand smoke exposure at home and at other places. In the hypothetical treatment, the isolation index was reduced by a fixed percentage or to the respective threshold value for those above the threshold to estimate the risk of lung cancer in less segregated scenarios. Threshold values were at the third quartile, the median, and the first quartile value of the population. In the fixed percentage reduction method, the isolation index was lowered by 1% or 5% annually. The risk under hypothetical settings were compared with the natural course, where the isolation index remained as observed.

**Figure 3.**
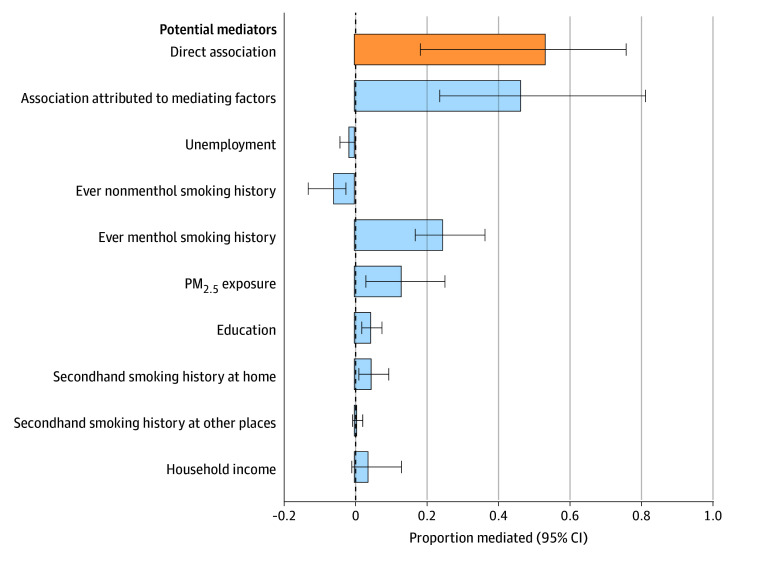
Estimated Proportion of the Association Between Isolation Index and Lung Cancer Incidence in African American Individuals PM_2.5_ indicates particulate matter with a diameter of 2.5 μm or less.

To decompose the isolation index–lung cancer association, we estimated the proportion mediated jointly and by each mediator individually. The IORW model showed that candidate mediators jointly contributed 46.6% (95% CI, 23.9% to 81.5%) of the observed association with lung cancer incidence in African American participants. The MSMs with IPW model analyzed the association mediated by individual mediators and found that 24.7% (95% CI, 17.1% to 36.6%) of the association was mediated by whether patients mainly smoked menthol cigarettes, 13.1% (95% CI, 3.2% to 25.4%) by PM_2.5_ exposure, 4.6% (95% CI, 2.1% to 7.7%) by education, 4.7% (95% CI, 1.3% to 9.6%) by exposure to secondhand smoke at home (Figure 3). Among African American participants, we estimated that residential segregation, as measured by the isolation index, was directly associated with 53.4% (95% CI, 18.5% to 76.1%) of the observed lung cancer incidence. Although nonmenthol smoking was associated with an increased risk of lung cancer, African American participants living in more segregated areas were less likely to primarily use nonmentholated cigarettes. Therefore, nonmentholated smoking status partially offset the association of the isolation index with lung cancer incidence by 5.8% (95% CI, 2.4% to 12.9%). The associations contributed by unemployment (−1.5%; 95% CI, −4.0% to 0.3%), exposure to secondhand smoke at other places (0.6%; 95% CI, −0.5% to 2.4%), and household income (3.8%; 95% CI, −0.7% to 13.2%) were not statistically significant, with the 95% CI encompassing zero.

## Discussion

This study is among the first to evaluate the impact of residential segregation on lung cancer risk while assessing mediating factors.^5^ We found that lowering the isolation index in hypothetical scenarios was associated with a significantly reduced number of lung cancer cases in African American but not non-Hispanic White participants. We further developed a mediation model to estimate factors underlying the association between residential segregation and lung cancer risk.

Although the literature has documented increased rates of advanced-stage lung cancer diagnosis,^15,39^ less surgical resection for early-stage disease, and increased lung cancer mortality^16^ and all-cause mortality^40^ in African Americans living in highly segregated areas, there has been limited evaluation of the link between residential segregation and lung cancer development. It is well established that lung cancer risk is driven by environmental toxins and associated with low social resources.^41,42,43^ Because residential segregation increases exposure to environmentally linked cancer-causing agents while concentrating poverty and decreasing social capital and investments, it clusters multicomponent causes of lung cancer risk in areas where socioeconomically marginalized communities are relegated.

Our study also focused on identifying mediators between residential segregation and lung cancer risk in African American individuals. We found that mentholated smoking status, PM_2.5_ exposure, secondhand smoking exposure at home, and education collectively contributed nearly 47% of the association. However, 53% of the variation in lung cancer risk in African American participants due to residential segregation remain unexplained, indicating the potential involvement of other significant factors.

African American communities experience increased targeted tobacco advertisement and marketing on billboards and print ads,^22^ and a recent study^23^ found that a higher proportion of African American individuals in a population correlates with increased tobacco retailers per square mile. The sale of mentholated cigarettes was also targeted to the African American community.^44^ These race-based targeting efforts contribute to higher primary and secondhand tobacco use in the African American community and to the subsequent development of lung cancer in this group, and thus, explain in part how both primary and secondhand tobacco exposure, particularly of mentholated cigarettes, mediates the association between residential segregation and lung cancer risk in African American individuals.^45^

Although racially segregated neighborhoods have elevated PM_2.5_ levels,^46^ it has not been previously demonstrated that residential segregation through PM_2.5_ exposure affects lung cancer risk among African American people. Our findings clarify that PM_2.5_ is associated with lung cancer risk among African American individuals in segregated areas, thus providing a modifiable target for policy and environmental justice efforts. Although this study is focused on the southern United States, increased exposure among African Americans to mentholated cigarettes, PM_2.5_, and residential segregation is pervasive throughout the United States, and thus, our findings are likely generalizable to regions outside of the US South.

Of note, we did not identify an association between lung cancer incidence and residential segregation in non-Hispanic White participants. Previous studies investigating segregation’s effects on lung cancer mortality rates found that increased segregation was associated with lower lung cancer mortality rates in non-Hispanic White individuals and higher ones for African American individuals.^16^ These results are indicative of the uneven distribution of resources and toxin exposure that African American and non-Hispanic White communities experience due to segregation—namely that racial segregation leads to more advantage for non-Hispanic White individuals while leading to disadvantage for African American individuals.

We found that lowering isolation index in hypothetical scenarios was associated with a reduced number of lung cancer cases among African American adults. This raises the question of how these scenarios might translate into real-world cancer prevention strategies. Numerous programs, legislation, and policies have been developed to make housing more affordable, make housing policies less discriminatory, or improve housing choice for historically excluded groups.^47^ However, none of these initiatives have had specific segregation improvement goals or targetable metrics that could lead to the prevention of cancer. Many public policies that are intended to address residential segregation are actually successful in moving individual households to less disadvantaged neighborhoods. And although these initiatives have had important health benefits,^48,49^ they are limited to individual household level impacts rather than lowering segregation at the population level. Thus, there is a major opportunity to integrate forward-thinking public policies that truly address segregation with scientifically validated segregation indices goals that aim to prevent lung cancer. Local initiatives that influence real isolation index value changes through a combination of legislation, zoning policies, and local programs may lead to novel and needed impact.

While we work on reducing segregation, it is crucial to mitigate the impact of mediators that lead to poor health through equity-focused interventions that lower exposure to cancer causing agents (eg, increasing green space and upholding stricter air quality regulations that lower PM_2.5_ levels; creating no-smoking policies in multiunit housing, passing legislature that abolishes mentholated tobacco advertisements and sales, and providing free smoking cessation groups). These and other mediator-targeted interventions could serve as a starting point for lowering lung and other cancer risk for African American communities while the longer-term goal of equity in housing and zoning policies that revitalize neglected, unhealthy communities is established.

### Strengths and Limitations

The study’s strengths include use of a large cohort that comprises medically underserved individuals; use of the isolation index, which strongly patterns unhealthy environments and exposures; and the assessment of the mechanisms linking residential segregation to lung cancer risk.^50^ Parametric g-computation handles time-varying exposures and confounders effectively.

However, limitations exist. First, neighborhood-level covariates and the isolation index were determined with baseline address, neglecting changes over time. Crucial factors, such as food access, diet, physical activity, smoking cessation education, and stress, were not included due to data limitations. Furthermore, the study is observational and causal inference is difficult to obtain.

## Conclusions

In this cohort study of non-Hispanic White and African American individuals from 12 US southern states, lower residential segregation was associated with a reduced number of lung cancer cases among African American but not non-Hispanic White individuals. This study suggests the need for urgent policy addressing structural racism and its mediators that prevent lung cancer.

## Notes

Supplement 1.
eMethods.
**eTable 1.** Association Between Lung Cancer Incidence and Candidate Mediators **eTable 2.** Demographic Characteristics and Outcome Stratified by Isolation Index Quartiles Among African American Participants **eTable 3.** Demographic Characteristics and Outcome Stratified by Isolation Index Quartiles Among non-Hispanic White Participants

Supplement 2.
Data Sharing Statement

## References

[1] Yang R, Cheung MC, Byrne MM, . Do racial or socioeconomic disparities exist in lung cancer treatment? Cancer. 2010;116(10):2437-2447. doi:10.1002/cncr.24986 20209616

[2] DeSantis CE, Miller KD, Goding Sauer A, Jemal A, Siegel RL. Cancer statistics for African Americans, 2019. CA Cancer J Clin. 2019;69(3):211-233. doi:10.3322/caac.21555 30762872

[3] American Cancer Society. Cancer facts & figures for African American/Black People 2022-2024. Accessed April 19, 2024. https://www.cancer.org/content/dam/cancer-org/research/cancer-facts-and-statistics/cancer-facts-and-figures-for-african-americans/2022-2024-cff-aa.pdf

[4] Williams DR, Collins C. Racial residential segregation: a fundamental cause of racial disparities in health. Public Health Rep. 2001;116(5):404-416. doi:10.1016/S0033-3549(04)50068-7 12042604 PMC1497358

[5] Landrine H, Corral I, Lee JGL, Efird JT, Hall MB, Bess JJ. Residential segregation and racial cancer disparities: a systematic review. J Racial Ethn Health Disparities. 2017;4(6):1195-1205. doi:10.1007/s40615-016-0326-9 28039602

[6] Egede LE, Walker RJ, Williams JS. addressing structural inequalities, structural racism, and social determinants of health: a vision for the future. J Gen Intern Med. 2024;39(3):487-491. doi:10.1007/s11606-023-08426-7 37740168 PMC10897090

[7] Dyer Z, Alcusky MJ, Galea S, Ash A. Measuring the enduring imprint of structural racism on American neighborhoods. Health Aff (Millwood). 2023;42(10):1374-1382. doi:10.1377/hlthaff.2023.00659 37782878 PMC10804769

[8] Bassett MT. Racial residential segregation, redlining, and health. JAMA Intern Med. 2024;184(11):1337-1338. doi:10.1001/jamainternmed.2024.5011 39348108

[9] Bailey ZD, Feldman JM, Bassett MT. How structural racism works—racist policies as a root cause of U.S. racial health inequities. N Engl J Med. 2021;384(8):768-773. doi:10.1056/NEJMms2025396 33326717 PMC11393777

[10] Namin S, Xu W, Zhou Y, Beyer K. The legacy of the Home Owners’ Loan Corporation and the political ecology of urban trees and air pollution in the United States. Soc Sci Med. 2020;246:112758. doi:10.1016/j.socscimed.2019.112758 31884239

[11] Ribeiro AI, Amaro J, Lisi C, Fraga S. Neighborhood socioeconomic deprivation and allostatic load: a scoping review. Int J Environ Res Public Health. 2018;15(6):1092. doi:10.3390/ijerph15061092 29843403 PMC6024893

[12] Rothstein R. The Color of Law: A Forgotten History of How Our Government Segregated America. Liveright Publishing Corporation; 2017.

[13] Zuk M, Bierbaum A, Chapple K, Thomas T. What are gentrification and displacement. Urban Displacement Project. Accessed May 28, 2025. https://www.urbandisplacement.org/about/what-are-gentrification-and-displacement

[14] Leibbrand C, Massey C, Alexander JT, Genadek KR, Tolnay S. The Great Migration and residential segregation in American cities during the twentieth century. Soc Sci Hist. 2020;44(1):19-55. doi:10.1017/ssh.2019.46 32546874 PMC7297198

[15] Annesi CA, Poulson M, Mak KS, . The impact of residential racial segregation on non-small cell lung cancer treatment and outcomes. Ann Thorac Surg. 2021;113(4):1291-1298. doi:10.1016/j.athoracsur.2021.04.096 34033745

[16] Hayanga AJ, Zeliadt SB, Backhus LM. Residential segregation and lung cancer mortality in the United States. JAMA Surg. 2013;148(1):37-42. doi:10.1001/jamasurgery.2013.408 23324839

[17] Johnson AM, Johnson A, Hines RB, Bayakly R. The effects of residential segregation and neighborhood characteristics on surgery and survival in patients with early-stage non-small cell lung cancer. Cancer Epidemiol Biomarkers Prev. 2016;25(5):750-758. doi:10.1158/1055-9965.EPI-15-1126 27197137

[18] Massey DS, Denton NA. The dimensions of residential segregation. Soc Forces. 1988;67(2):281-315. doi:10.2307/2579183

[19] Chang VW. Racial residential segregation and weight status among US adults. Soc Sci Med. 2006;63(5):1289-1303. doi:10.1016/j.socscimed.2006.03.049 16707199

[20] Motairek I, Chen Z, Makhlouf MHE, Rajagopalan S, Al-Kindi S. Historical neighborhood redlining and contemporary environmental racism. Local Environ. 2023;28(4):518-528. doi:10.1080/13549839.2022.2155942 37588138 PMC10427113

[21] Woo B, Kravitz-Wirtz N, Sass V, Crowder K, Teixeira S, Takeuchi DT. Residential segregation and racial/ethnic disparities in ambient air pollution. Race Soc Probl. 2019;11(1):60-67. doi:10.1007/s12552-018-9254-0 31440306 PMC6706065

[22] Primack BA, Bost JE, Land SR, Fine MJ. Volume of tobacco advertising in African American markets: systematic review and meta-analysis. Public Health Rep. 2007;122(5):607-615. doi:10.1177/003335490712200508 17877308 PMC1936959

[23] Kong AY, Delamater PL, Gottfredson NC, Ribisl KM, Baggett CD, Golden SD. Sociodemographic inequities in tobacco retailer density: do neighboring places matter? Health Place. 2021;71:102653. doi:10.1016/j.healthplace.2021.102653 34461529 PMC8490323

[24] Wailoo K. Pushing Cool: Big Tobacco, Racial Marketing, and the Untold Story of the Menthol Cigarette. University of Chicago Press; 2021.

[25] Alcaraz KI, Wiedt TL, Daniels EC, Yabroff KR, Guerra CE, Wender RC. Understanding and addressing social determinants to advance cancer health equity in the United States: a blueprint for practice, research, and policy. CA Cancer J Clin. 2020;70(1):31-46. doi:10.3322/caac.21586 31661164

[26] Signorello LB, Hargreaves MK, Steinwander MD, et al. Southern Community Cohort Study: establishing a cohort to investigate health disparities. J Natl Med Assoc. 2005;97(7):972–979. PMC2569308 16080667

[27] Signorello LB, Hargreaves MK, Blot WJ. The Southern Community Cohort Study: investigating health disparities. J Health Care Poor Underserved. 2010;21(1)(suppl):26-37. doi:10.1353/hpu.0.0245 20173283 PMC2940058

[28] Dai D. Black residential segregation, disparities in spatial access to health care facilities, and late-stage breast cancer diagnosis in metropolitan Detroit. Health Place. 2010;16(5):1038-1052. doi:10.1016/j.healthplace.2010.06.012 20630792

[29] Southern Community Cohort Study. Accessed May 28, 2025. https://www.southerncommunitystudy.org/

[30] Meng J, Li C, Martin RV, van Donkelaar A, Hystad P, Brauer M. Estimated long-term (1981-2016) Concentrations of ambient fine particulate matter across North America from chemical transport modeling, satellite remote sensing, and ground-based measurements. Environ Sci Technol. 2019;53(9):5071-5079. doi:10.1021/acs.est.8b06875 30995030

[31] VanderWeele TJ, Robinson WR. On the causal interpretation of race in regressions adjusting for confounding and mediating variables. Epidemiology. 2014;25(4):473-484. doi:10.1097/EDE.0000000000000105 24887159 PMC4125322

[32] Smith TJS, Keil AP, Buckley JP. Estimating causal effects of interventions on early-life environmental exposures using observational data. Curr Environ Health Rep. 2023;10(1):12-21. doi:10.1007/s40572-022-00388-y 36418665 PMC11975414

[33] Breskin A, Edmonds A, Cole SR, . G-computation for policy-relevant effects of interventions on time-to-event outcomes. Int J Epidemiol. 2021;49(6):2021-2029. doi:10.1093/ije/dyaa156 33141177 PMC7825964

[34] Li S, Karagas MR, Jackson BP, Passarelli MN, Gui J. Adaptive-mixture-categorization (AMC)-based g-computation and its application to trace element mixtures and bladder cancer risk. Sci Rep. 2022;12(1):17841. doi:10.1038/s41598-022-21747-7 36284198 PMC9596719

[35] Chen C, Chen H, van Donkelaar A, . Using parametric g-computation to estimate the effect of long-term exposure to air pollution on mortality risk and simulate the benefits of hypothetical policies: the Canadian Community Health Survey Cohort (2005 to 2015). Environ Health Perspect. 2023;131(3):37010. doi:10.1289/EHP11095 36920446 PMC10016347

[36] Bonner SN, Curley R, Love K, Akande T, Akhtar A, Erhunmwunsee L. Structural Racism and Lung Cancer Risk: A Scoping Review. JAMA Oncol. 2024;10(1):122-128. doi:10.1001/jamaoncol.2023.4897 38032677

[37] Nandi A, Vanderweele TJ. Mediation Analysis in Social Epidemiology: Methods in Social Epidemiology. Jossey-Bass; 2017.

[38] Nguyen QC, Osypuk TL, Schmidt NM, Glymour MM, Tchetgen Tchetgen EJ. Practical guidance for conducting mediation analysis with multiple mediators using inverse odds ratio weighting. Am J Epidemiol. 2015;181(5):349-356. doi:10.1093/aje/kwu278 25693776 PMC4339385

[39] Haas JS, Earle CC, Orav JE, Brawarsky P, Neville BA, Williams DR. Racial segregation and disparities in cancer stage for seniors. J Gen Intern Med. 2008;23(5):699-705. doi:10.1007/s11606-008-0545-9 18338215 PMC2324162

[40] Joshi A, Wilson LE, Pinheiro LC, Judd S, Akinyemiju T. Association of racial residential segregation with all-cause and cancer-specific mortality in the Reasons for Geographic and Racial Differences in Stroke (REGARDS) cohort study. SSM Popul Health. 2023;22:101374. doi:10.1016/j.ssmph.2023.101374 37132018 PMC10149269

[41] Sidorchuk A, Agardh EE, Aremu O, Hallqvist J, Allebeck P, Moradi T. Socioeconomic differences in lung cancer incidence: a systematic review and meta-analysis. Cancer Causes Control. 2009;20(4):459-471. doi:10.1007/s10552-009-9300-8 19184626

[42] Luo J, Hendryx M, Ducatman A. Association between six environmental chemicals and lung cancer incidence in the United States. J Environ Public Health. 2011;2011:463701. doi:10.1155/2011/463701 21776439 PMC3136160

[43] Berg CD, Schiller JH, Boffetta P, ; International Association for the Study of Lung Cancer (IASLC) Early Detection and Screening Committee. Air pollution and lung cancer: a review by International Association for the Study of Lung Cancer Early Detection and Screening Committee. J Thorac Oncol. 2023;18(10):1277-1289. doi:10.1016/j.jtho.2023.05.024 37277094

[44] Mills SD, Henriksen L, Golden SD, . Disparities in retail marketing for menthol cigarettes in the United States, 2015. Health Place. 2018;53:62-70. doi:10.1016/j.healthplace.2018.06.011 30055469 PMC6161357

[45] Landrine H, Klonoff EA. Racial segregation and cigarette smoking among Blacks: findings at the individual level. J Health Psychol. 2000;5(2):211-219. doi:10.1177/135910530000500211 22049011

[46] Yitshak-Sade M, Lane KJ, Fabian MP, . Race or racial segregation? modification of the PM2.5 and cardiovascular mortality association. PLoS One. 2020;15(7):e0236479. doi:10.1371/journal.pone.0236479 32716950 PMC7384646

[47] Steil J, Lens M. Public policies to address residential segregation and improve health. Health Affairs. April 27, 2023. Accessed May 28, 2025. https://www.healthaffairs.org/content/briefs/public-policies-address-residential-segregation-and-improve-health

[48] Sanbonmatsu L, Ludwig J, Katz LF. Moving to Opportunity for Fair Housing Demonstration Program: final impacts evaluation. US Department of Housing and Urban Development. November 2011. Accessed May 28, 2025. https://www.huduser.gov/publications/pdf/mtofhd_fullreport_v2.pdf

[49] Chetty R, Hendren N, Katz LF. The effects of exposure to better neighborhoods on children: new evidence from the Moving to Opportunity experiment. *Am Econ Assoc*. 2016;106(4):855-902. Accessed May 28, 2025. https://www.aeaweb.org/articles?id=10.1257/aer.20150572 10.1257/aer.20150572 29546974

[50] Kramer MR, Hogue CR. Is segregation bad for your health? Epidemiol Rev. 2009;31:178-194. doi:10.1093/epirev/mxp001 19465747 PMC4362512

