# Supplementary material for: Residential Segregation and Lung Cancer Risk in African American Adults
Source: JAMA Netw Open. 2025 Jul 1;8(7):e2518481. doi: 10.1001/jamanetworkopen.2025.18481 (PMC12215573; doi:10.1001/jamanetworkopen.2025.18481)
Supplement: Supplement 1. — eMethods. eTable 1. Association Between Lung Cancer Incidence and Candidate Mediators eTable 2. Demographic Characteristics and Outcome Stratified by Isolation Index Quartiles Among African American Participants eTable 3. Demographic Characteristics and Outcome Stratified by Isolation Index Quartiles Among non-Hispanic White Participants [file jamanetwopen-e2518481-s001.pdf]

Supplemental Online Content

Xiao Y, Zou X, Tribby CP, et al. Residential segregation and lung cancer risk in African American adults. *JAMA Netw Open.* 2025;8(7):e2518481.
doi:10.1001/jamanetworkopen.2025.18481

eMethods.

eTable 1. Association Between Lung Cancer Incidence and Candidate Mediators

eTable 2. Demographic Characteristics and Outcome Stratified by Isolation Index Quartiles Among African American Participants

eTable 3. Demographic Characteristics and Outcome Stratified by Isolation Index Quartiles Among non-Hispanic White Participants

This supplemental material has been provided by the authors to give readers additional information about their work.

© 2025 Xiao Y et al. *JAMA Network Open*.

eMethods.

1. Study Subject

Participants were enrolled from 12 US southern states including Alabama, Arkansas, Florida, Georgia, Kentucky, Louisiana, Mississippi, North Carolina, South Carolina, Tennessee, Virginia, West Virginia.

2. Exposure

Isolation index is calculated using the formula: $I_j = \sum_{i=1}^{n}(x_i/X_j)(x_i/t_i)$ where j denotes the census tract, i represents the block group. x_i is the AA population in block i, X_j is the total AA population in the census tract j, t_i is the total population in i and n is the number of block within j. x_i/X_j evaluates the concentration of AA population within a block compared to the census tract and x_i/t_i examines the proportion of AA individuals among all racial groups within the block.

3. Details of Mediation Analysis

Marginal structural models (MSMs) with inverse propensity weighting (IPW) assessed mediating effects individually. First, to eliminate confounder-exposure association, we conducted logistic regressions to estimate the exposure IPW, which is the inverse probability of receiving the observed exposure conditional on exposure-outcome confounders. The exposure isolation index was dichotomized at the median. Second, we calculated mediator IPW to remove links between the examined mediator, confounders, and other potential mediators. Finally, we estimated total effect (TE), natural direct effect (NDE) and natural indirect effect (NIE) of isolation index through individual mediators. NDE can be estimated with a regression model of lung cancer as a function of isolation index and the examined mediator, weighted by the product of exposure IPW and mediator IPW. To calculate the NIE, we fitted a regression model of the examined mediator as a function of isolation index weighted by exposure IPW to estimate the effect of the exposure on a mediator. NIE was identified as the product of isolation index effect on a mediator and the mediator effect on the lung cancer risk obtained from the first model. As we estimate risks on the absolute scale, TE was the sum of NIE and NDE. Proportions of effect mediated through candidate mediators were calculated as NIE divided by TE.

We then used inverse odds ratio weighting (IORW) to analyze the joint effect of all potential mediators. We first fitted a logistic regression with exposure, covariates, all candidate mediators, and an interaction term of PM2.5 and year of follow-up from 2002 to 2019. We then used the inverse exposure-mediator odds ratio as weight to estimate the NDEs. The total effect was estimated using a standard logistic regression without the weight. The joint effect of mediators was the difference between the total effect and natural direct effect. The 95% confidence intervals for TE, NIE and NDE were calculated from 100 bootstrap iterations.

© 2025 Xiao Y et al. *JAMA Network Open*.

eTable 1. Association Between Lung Cancer Incidence and Candidate Mediators

	Crude Model		Adjusted Model	
Variable	HR or Pooled OR[1] (95% CI)	p-value[2]	HR or Pooled OR[1] (95% CI)	p-value[2]
	**African American**			
**PM$_{2.5}$**	1.11 (1.06-1.16)	< .001	1.07 (1.02-1.12)	.002
**Secondhand Smoking History at Home**	1.55 (1.38-1.75)	< .001	1.12 (1.01-1.24)	.03
**Secondhand Smoking History at Other Places**	1.20 (1.07-1.35)	.003	1.11 (0.98-1.26)	.10
**Menthol Smoking History**	2.11 (1.86-2.40)	< .001	6.5 (5.13-8.24)	< .001
**Nonmenthol Smoking History**	2.90 (2.53-3.32)	< .001	11.49 (8.87-14.88)	< .001
**Education (Ref: College and above)**				
HS or below college	1.69 (1.45-1.96)	< .001	1.31 (1.11-1.54)	< .001
Less than 9 years	2.72 (2.2-3.35)	< .001	2.01 (1.6-2.52)	< .001
**Household Income (Ref** $50,000 and more)				
At least $25,000 but less than $50,000	1.34 (0.87-2.07)	.18	1.08 (0.67-1.72)	.76
At least $15,000 but less than $25,000	2.14 (1.44-3.19)	< .001	1.49 (0.96-2.3)	.07
Less than $15,000	2.74 (1.87-4.02)	< .001	1.61 (1.05-2.47)	.03
**Employment Status (Ref** employed**)**				
Unemployed	1.71 (1.51-1.94)	<.001	1.24 (1.08-1.42)	.002
	**Non-Hispanic White**			
**PM$_{2.5}$**	1.12 (1.05-1.18)	.001	1.09 (1.03-1.15)	.004
**Secondhand Smoking History at Home**	1.93 (1.62-2.28)	< .001	1.16 (0.97-1.39)	.11
**Secondhand Smoking History at Other Places**	1.1 (0.92-1.31)	.30	0.95 (0.79-1.14)	.55
**Menthol Smoking History**	1.21 (0.99-1.49)	.07	7.89 (4.96-12.56)	< .001
**Nonmenthol Smoking History**	3.56 (2.94-4.32)	< .001	10.98 (7.12-16.93)	< .001
**Education (Ref: College and above)**				
HS or below college	1.83 (1.5-2.22)	< .001	1.19 (0.95-1.48)	.13
Less than 9 years	2.79 (2.11-3.69)	< .001	1.52 (1.11-2.08)	.01
**Household Income (Ref** $50,000 and more)				
At least $25,000 but less than $50,000	1.66 (1.09-2.53)	.02	1.15 (0.73-1.82)	.54
At least $15,000 but less than $25,000	2.78 (1.9-4.06)	< .001	1.65 (1.08-2.51)	.02
Less than $15,000	4.05 (2.87-5.71)	< .001	2.09 (1.4-3.12)	< .001
**Employment Status (Ref** employed**)**				
Unemployed	2.24 (1.85-2.71)	< .001	1.5 (1.21-1.87)	< .001
	**All**			
**PM$_{2.5}$**	1.08 (1.03-1.13)	< .001	1.06 (1.01-1.11)	
**Secondhand Smoking History at Home**	1.66 (1.51-1.83)	< .001	1.12 (1.01, 1.24)	.02
**Secondhand Smoking History at Other Places**	1.16 (1.05-1.28)	.004	0.98 (0.89, 1.09)	.77
**Menthol Smoking History**	1.55 (1.41-1.70)	< .001	6.63 (5.37, 8.18)	< .001
**Nonmenthol Smoking History**	2.79 (2.53-3.07)	< .001	11.07 (8.94, 13.71)	< .001
**Education (Ref: College and above)**				
HS or below college	1.69 (1.5-1.9)	< .001	1.26 (1.1, 1.43)	< .001
Less than 9 years	2.71 (2.29-3.2)	< .001	1.82 (1.52, 2.19)	< .001
**Household Income (Ref** $50,000 and more)				
At least $25,000 but less than $50,000	1.43 (1.06-1.93)	.02	1.16 (0.84, 1.6)	0.37
At least $15,000 but less than $25,000	2.26 (1.73-2.95)	< .001	1.65 (1.23, 2.21)	< .001
Less than $15,000	2.99 (2.32-3.85)	< .001	1.87 (1.4, 2.49)	< .001
**Employment Status (Ref** employed**)**				
Unemployed	1.86 (1.67-2.07)	< .001	1.31 (1.17, 1.47)	< .001

[1] Pooled OR = Pooled Odds Ratio in unadjusted model or adjusted for PM$_{2.5}$, HR = Hazard Ratio for menthol and non-menthol smoking status, secondhand smoking history at home or at other places, education, household income and unemployment. CI = Confidence Interval.

© 2025 Xiao Y et al. *JAMA Network Open*.

[2]P-value obtained from Cox regression or pooled logistic regression.

Note: An OR or HR greater than 1 indicates that a one-unit increase in the continuous variable or belonging to a specific group for the categorical variable is associated with an increase in the risk of developing lung cancer.

For both AAs and NHWs, higher $PM_{2.5}$ and having a history of menthol or non-menthol smoking, receiving lower education and income are significantly associated with higher risk of lung cancer. Exposure to secondhand smoke at home and other places is also associated with higher risk of lung cancer in the crude model. Exposure to secondhand smoke at home was associated with higher lung cancer risk, but not significantly in adjusted model within the NHW subgroups. Similarly, exposure to secondhand smoke in other places showed no significant association after adjusting for other variables for AAs and NHWs.

© 2025 Xiao Y et al. *JAMA Network Open*.

eTable 2. Demographic Characteristics and Outcome Stratified by Isolation Index Quartiles Among African American Participants

Characteristics	First Quartile (Least Segregated), N = 12,725	Second Quartile, N = 12,725	Third Quartile, N = 12,724	Fourth Quartile (most segregated), N = 12,724
**Sex, No. (%)**				
Female	7,682 (60.4)	7,733 (60.8)	7,359 (57.8)	6,697 (52.6)
Male	5,043 (39.6)	4,992 (39.2)	5,365 (42.2)	6,027 (47.4)
**Age, median (IQR), y**	50 (45, 56)	50 (45, 57)	50 (44, 56)	49 (44, 55)
**$PM_{2.5}$, median (IQR), $\mu g/m^3$**	10.3 (9.3, 11.5)	10.3 (9.6, 11.6)	11.1 (9.8, 12.1)	11.8 (10.8, 12.5)
N/A, No. (%)	72(.6)	26(.2)	22(.2)	25(.2)
**Household Income, No. (%)**				
Less than $15,000	6,593 (51.8)	7,399 (58.1)	7,916 (62.2)	8,265 (65.0)
At least $15,000 but less than $25,000	2,940 (23.1)	2,943 (23.1)	2,698 (21.2)	2,704 (21.3)
At least $25,000 but less than $50,000	1,926 (15.1)	1,601 (12.6)	1,429 (11.2)	1,285 (10.1)
$50,000 and more	1,098 (8.6)	621 (4.9)	537 (4.2)	333 (2.6)
Not Reported	168 (1.3)	161 (1.3)	144 (1.1)	137 (1.1)
**Education, No. (%)**				
Less than 9 years	853 (6.7)	1,158 (9.1)	1,080 (8.5)	922 (7.2)
High School or below college	7,640 (60.0)	8,027 (63.1)	8,110 (63.7)	8,566 (67.3)
College and above	4,221 (33.2)	3,529 (27.7)	3,529 (27.7)	3,232 (25.4)
Not Reported	11 (.1)	11 (.1)	5 (< .1)	4 (< .1)
**Smoking status, No. (%)**				
Never smoking	5,228 (41.1)	5,180 (40.7)	4,613 (36.3)	3,853 (30.3)
Primarily menthol smoking	5,943 (46.7)	6,197 (48.7)	6,825 (53.6)	7,741 (60.8)
Primarily nonmenthol smoking	1,512 (11.9)	1,304 (10.2)	1,248 (9.8)	1,100 (8.6)
Not Reported	42 (.3)	44 (.3)	38 (.3)	30 (.2)
**Secondhand Smoke Exposure at Home, No. (%)**				
No	8,548 (67.2)	8,293 (65.2)	7,965 (62.6)	7,334 (57.6)
Yes	3,775 (29.7)	4,021 (31.6)	4,384 (34.5)	5,006 (39.3)
Not Reported	402 (3.2)	411 (3.2)	375 (2.9)	384 (3.0)
**Secondhand Smoke Exposure at Other places, No. (%)**				
No	7,732 (60.8)	7,626 (59.9)	7,346 (57.7)	6,978 (54.8)
Yes	4,540 (35.7)	4,652 (36.6)	4,970 (39.1)	5,291 (41.6)
Not Reported	453 (3.6)	447 (3.5)	408 (3.2)	455 (3.6)
**Lung cancer incidence, No. (%)**	7,732 (60.8)	7,626 (59.9)	7,346 (57.7)	6,978 (54.8)

© 2025 Xiao Y et al. *JAMA Network Open*.

eTable 3. Demographic Characteristics and Outcome Stratified by Isolation Index Quartiles Among non-Hispanic White Participants

Characteristic	First Quartile (Least Segregated), N = 5,184	Second Quartile, N = 5,184	Third Quartile, N = 5,184	Fourth Quartile (most segregated), N = 5,184
**Sex, No. (%)**				
	3,251 (62.7)	3,208 (61.9)	3,163 (61.0)	2,939 (56.7)
**Female**				
**Male**	1,933 (37.3)	1,976 (38.1)	2,021 (39.0)	2,245 (43.3)
**Age, median (IQR), y**	54 (47, 61)	52 (46, 59)	51 (46, 59)	51 (45, 58)
**$PM_{2.5}$, median (IQR), µg/m^3**	9.4 (8.4, 10.3)	10.2 (9.2, 11.2)	10.4 (9.3, 11.5)	10.6 (9.6, 11.9)
N/A, **No. (%)**	115 (2.2)	57 (1.1)	19 (.4)	8 (.2)
**Household Income, No. (%)**				
**Less than $15,000**	2,020 (39.0)	2,235 (43.1)	2,626 (50.7)	2,950 (56.9)
**At least $15,000 but less than $25,000**	923 (17.8)	989 (19.1)	983 (19.0)	967 (18.7)
**At least $25,000 but less than $50,000**	978 (18.9)	844 (16.3)	835 (16.1)	680 (13.1)
**$50,000 and more**	1,175 (22.7)	1,044 (20.1)	677 (13.1)	522 (10.1)
**Not Reported**	88 (1.7)	72 (1.4)	63 (1.2)	65 (1.3)
**Education, No. (%)**				
**Less than 9 years**	361 (7.0)	335 (6.5)	448 (8.6)	530 (10.2)
**High School or below college**	2,649 (51.1)	2,703 (52.1)	2,906 (56.1)	2,933 (56.6)
**College and above**	2,172 (41.9)	2,143 (41.3)	1,830 (35.3)	1,716 (33.1)
**Not reported**	2 (< .01)	3 (.1)	0 (.0)	5 (.1)
**Smoking status, No. (%)**				
Never smoking	2,054 (39.6)	1,766 (34.1)	1,562 (30.1)	1,561 (30.1)
Primarily menthol smoking	763 (14.7)	868 (16.7)	947 (18.3)	1,035 (20.0)
Primarily nonmenthol smoking	2,342 (45.2)	2,534 (48.9)	2,656 (51.2)	2,570 (49.6)
Not Reported	25 (.5)	16 (.3)	19 (.4)	18 (.3)
**Secondhand Smoke Exposure at Home, No. (%)**				
**No**	3,573 (68.9)	3,310 (63.9)	3,162 (61.0)	2,960 (57.1)
**Yes**	1,389 (26.8)	1,657 (32.0)	1,808 (34.9)	1,990 (38.4)
**Not Reported**	222 (4.3)	217 (4.2)	214 (4.1)	234 (4.5)
**Secondhand Smoke Exposure at Other places, No. (%)**				
**No**	3,320 (64.0)	3,266 (63.0)	3,155 (60.9)	2,988 (57.6)
**Yes**	1,609 (31.0)	1,676 (32.3)	1,785 (34.4)	1,932 (37.3)
**Not Reported**	255 (4.9)	242 (4.7)	244 (4.7)	264 (5.1)
**Lung cancer incidence, No. (%)**	119 (2.3)	140 (2.7)	153 (3.0)	154 (3.0)

© 2025 Xiao Y et al. *JAMA Network Open*.
