# Supplementary material for: Residential Segregation and Lung Cancer Risk in African American Adults
Source: JAMA Netw Open. 2025 Jul 1;8(7):e2518481. doi: 10.1001/jamanetworkopen.2025.18481 (PMC12215573; doi:10.1001/jamanetworkopen.2025.18481)
Supplement: Supplement 2. — Data Sharing Statement [file jamanetwopen-e2518481-s002.pdf]

Data Sharing Statement

Xiao. Residential Segregation and Lung Cancer Risk in African American Adults. *JAMA Netw Open*. Published July 01, 2025. doi:10.1001/jamanetworkopen.2025.18481

Data
Data available: Yes
Data types: Data dictionary
How to access data: on the SCCS website
When available: With publication

Supporting Documents
Document types: None

Additional Information
Who can access the data: researchers whose proposed use of the data has been approved.
Types of analyses: for any purpose approved by the SCCS team.
Mechanisms of data availability: after approval of a proposal
